# The time has come to eliminate the gaps in the under-recognized burden of elder mistreatment: A community-based, cross-sectional study from rural eastern Nepal

**DOI:** 10.1371/journal.pone.0198410

**Published:** 2018-06-20

**Authors:** Uday Narayan Yadav, Man Kumar Tamang, Grish Paudel, Bharat Kafle, Suresh Mehta, Varalakshmi Chandra Sekaran, Jeroen R. J. H Gruiskens

**Affiliations:** 1 Forum for Health Research and Development, Dharan, Nepal; 2 School of Public Health and Community Medicine, University of New South Wales, Sydney, Australia; 3 Central Campus of Technology, Tribhuvan University, Dharan, Nepal; 4 Central Department of Population Studies, Tribhuvan University, Kathmandu, Nepal; 5 Nepal Public Health Foundation, Kathmandu, Nepal; 6 Department of Community Medicine, Melaka Manipal Medical College, Manipal University, Manipal, India; 7 CAPHRI School of Public Health and Preventive Medicine, Maastricht University, Maastricht, The Netherlands; Sciensano, BELGIUM

## Abstract

**Background:**

Elder mistreatment is a well-recognized public health issue with complex underlying factors. The current study hypothesized that there is no effect of any of the following factors on any type of elder mistreatment: ethnicity, age group, education status, gender, living arrangement, concentration problems, medication for any disease, income level of caregiver, use of alcohol and tobacco products, and dependence on family or caregivers for daily activities.

**Materials and methods:**

We conducted a cross-sectional study of 339 elders adults aged 60 or above residing in a rural part of eastern Nepal between August and November 2016. Multi-stage cluster sampling was adopted to select the study subjects. Information was collected using semi-structured questionnaires administered to elderly people by a designated interviewer. Factors associated with elder mistreatment were analyzed using logistic regression.

**Results:**

Our findings revealed that 61.7% of 60+-year-olds experienced some form of mistreatment (physical 2.4%, psychological 22.4%, caregiver neglect 57.5%, financial 12.1% and stranger-inflicted 8.3%). Elder mistreatment was associated with the following characteristics of elders: dependent on family for daily living activities, illiterate, experiencing concentration problems, residing in a living arrangement with their son(s)/daughter(s)-in-law, taking regular medications, belonging to the Dalit community according to the Hindu traditional caste system, and residing with a caregiver having a monthly family income of less than NRs. 20,000 (193USD).

**Conclusions:**

Our data show that elder mistreatment is prevalent in a rural community of Nepal. Addressing the lower socio-economic or socio-cultural classes of caregivers and elders via community-focused development programs might have significant implications for improving the well-being of elders.

## Introduction

The worldwide demographic transition indicates a higher proportion of elderly people than children, which is projected to increase to approximately 1.5 billion elders by 2050, compared to the 254 million estimated in 2010. The major shift in the population is expected to occur mostly in developing countries [1]. This remarkable change is being attributed to a decrease in fertility rates and a decline in death rates as a result of successful public health interventions all over the world. Rapid gains have been seen in East Asia, where life expectancy at birth has increased to more than 74 years compared to less than 45 years in 1950 [2]. The 2011 census of Nepal showed that 8.1% of the country's total population constitutes elders [3]. Unlike high income countries, a majority of the elder population lives in the rural regions of Nepal.

The 2002 Madrid International Plan of Action on Aging (MIPAA) is the first global agreement recognizing the needs of elderly people and emphasizing that they should be included in policy in all developmental processes, thus advancing their health and well-being [1]. The dignity and fundamental rights of elders must be respected with a great sense of responsibility and without any discrimination by individuals, families, relatives, communities, and the government at all levels of public service delivery. However, it has been recognized that the circumstance is unprecedented in many respects in which inevitable challenges are faced by elders resulting from modernization, comorbidities, poor social and family support, inadequate economic support, and a lack of aging policies and community-based care systems, thereby reducing their quality of life [4]. These disadvantages may contribute to poorer quality of life as well as put elders at risk for various types of mistreatment, including physical, psychological or emotional, sexual, and financial mistreatment [5]. Epidemiological studies have reported global variations in the prevalence of mistreatment, ranging between 2.0% to 79.7% in terms of a pooled estimate from five continents and between 2.2% to 66% in Asia [6,7]. The results of a systematic review conducted by Dong revealed group estimates in Asia ranging from 14% in India to 36.2% in China. European estimates ranged from 2.2% in Ireland to 61.1% in Croatia, while the Americas showed estimates ranging from 10% in the USA to 79.7% in Peru [6]. The review published in Lancet in 2017 showed the pooled prevalence estimates for different subtypes of elder mistreatment: 11·6% (8·1–16·3) for psychological mistreatment, 6·8% (5·0–9·2) for financial mistreatment, 4·2% (2·1–8·1) for neglect, 2·6% (1·6–4·4) for physical mistreatment, and 0·9% (0·6–1·4) for sexual mistreatment [8]. A recently published Malaysian study reported a prevalence of 4.5% in the community setting [9]. A recent scoping review by Pillemer et al. showed that elderly adults’ functional dependence, ethnicity, gender, age, poor physical health, low income, and substance abuse are strongly associated with elder mistreatment; however, the findings mentioned social support and living arrangements as protective factors against elder mistreatment [10].

In the community/society, a large proportion of elder mistreatment is hidden from the law and general public; thus, an analogy to the hidden mass of the "iceberg phenomenon of the disease" can be made. Elder mistreatment “any type of action, series of actions or lack of actions, which produce physical or psychological harm (loneliness, negligence, non-responsiveness, exploitation from family members), and which is set within a relationship of trust or dependence” [11–13].

In this context, elder mistreatment is well documented in the Western world, and there are economic and social policies to address the issue. Nonetheless, in the context of developing countries, including Nepal, with a lack of adequate research in this niche area, documentation is limited. Elder issues are not considered a major issue needing attention, given that the Nepalese cultural milieu is bound by strong family ties. However, there is a growing need to shift thinking towards issues faced by elders due to growing generation gaps, the mobility of economically active people for jobs and better education, adjustment problems, disrespect by the younger generation and abusive behavior from family members and caregivers, all of which have affected the lives of elders [9,10,13]. In a survey of 212 elderly people in urban Nepal, the rate of some form of elder mistreatment was found to be 49.1% [13]. Similarly, a baseline study on reported cases of elder mistreatment in the Nepali press showed the presence of physical mistreatment (43%) and neglect (33.3%), as well as mistreatment leading to the death of elder victims in 49% of cases [5]. Additionally, the same report illustrated that urban areas have more reported cases of mistreatment than other regions, where poverty, family conflict/jealousy/greed, and corrupt attitudes were reflected as prime causes of negligence towards elders [14]

The government of Nepal has formulated a national policy on aging in the form of various acts on property rights distribution, social security and access to health facilities. Nevertheless, defined rules or acts against elder mistreatment is lacking for the burgeoning elder population. Given that elder mistreatment can have a range of negative outcomes, such as psychological disorders and an increased risk for death and suicide [15], understanding associated factors and preventing elder mistreatment should be top research priorities in developing countries, including Nepal. The emphasis on elder mistreatment is appropriate because it is a widespread problem among elderly people that is largely preventable. Therefore, a better understanding of this problem and its risk factors should be a priority in national policies and strategies. There have been very few studies in varied settings of Nepal [5,13]. Building on the existing literature, the present study attempts to bridge a critical knowledge gap in this field by examining the prevalence of elder mistreatment and associated factors among elders in rural communities. In this study, we hypothesized (null hypothesis) that there is no effect of ethnicity, age group, education status, gender, living arrangement, concentration problems, medication for any disease, income level of caregiver, use of alcohol and tobacco products, and dependence on family or caregivers for daily activities on any type of elder mistreatment.

## Materials and methods

### Study designs and participants

**Fig 1:**
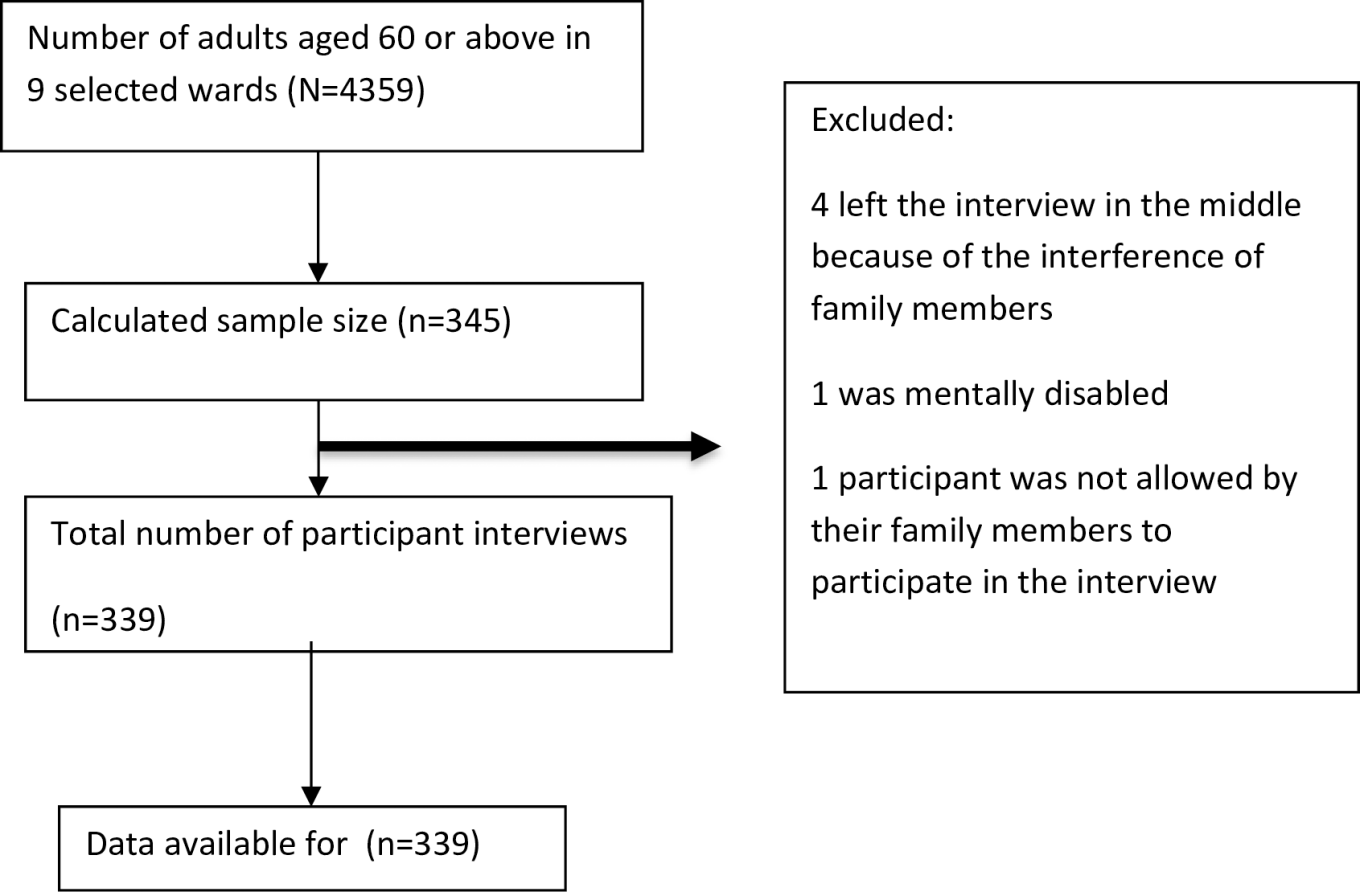
Fig illustrates the flow diagram of the study subjects based on the STrengthening the Reporting of OBservational studies in Epidemiology (STROBE) guidelines [16].

This is a community-based cross-sectional study among elderly adults aged 60 or above in the Morang District of eastern Nepal between August and November 2016. A multi-stage cluster sampling approach was adopted to select the study subjects. The sample size of 345 was based on following assumptions: prevalence = 10.8% [13], sampling error = 5.0%, CI = 95.0%, design effect = 2 and non-response rate = 15.0%. We approached 345 participants, of whom only 339 participated in the study, leading to a response rate of 98.26% (Fig 1). In the first stage, three village development committees (VDCs) were randomly selected from the list of 50 VDCs within the Morang District. Of the three VDCs, one was dominated by people of mixed origin (of plain origin and of hilly origin), while the other two VDCs were mainly dominated by people of plain origin, mostly involved in agriculture. Second, three wards were randomly selected in each of the selected VDCs. Finally, individuals were selected randomly from the list of eligible subjects provided by the VDC secretary and were interviewed by the trained interviewers in the community setting. The eligible study population included adults aged 60 years or above who had lived in the community for the past year and were Nepali nationals. The exclusion criteria included residing in care institutions, being mentally disabled, being seriously ill, having a hearing disability or being unable to communicate. We defined mental disability as “cerebral palsy or mental retardation” that begins at birth or at any stage of life.

### Data collection and study variables

Data were collected with the use of a 21-item interviewer-administered semi-structured tool developed after a literature review, pre-testing and expert validation. The tool was designed because previously published tools were insufficient in capturing mistreatment cases in the Nepalese cultural setting. The reliability was measured by using Cronbach's alpha (0.75). Information on mistreatment was collected by probing questions on the different types of mistreatment participants may have experienced in last three months from caregivers or family members. Competency-based hands-on training (two days) for enumerators was provided in a standardized manner focusing on the following: study methodology, components of questionnaires and their importance, and the method of collecting precise information from the study participants. The enumerators were bachelor-level health graduates with training in the health sciences after twelve years of schooling. The technical training was provided on record keeping, documentation and quality control. Ethical approval was obtained from the Nepal Health Research Council, Ministry of Health, Government of Nepal (194/2016). Prior to the interview, written informed consent was obtained from all literate participants, and thumb impressions were obtained from illiterate participants. To comply with data privacy protection, the personal data of every participant were anonymized.

### Dependent variables

The experience of any mistreatment within the last three months among adults aged 60 years or above was the primary outcome variable in this study, and self-reported measures (during past three months) were taken into account. The answer choices were *yes/no*.

For subtypes of elder mistreatment, respondents were asked questions related to the following types of mistreatment: *physical mistreatment*, or acts carried out with an intention to cause physical injury or pain; *psychological mistreatment*, or acts carried out with an intention of causing emotional pain; *caregiver neglect*, or negligence, non-responsiveness and lack of fulfillment of basic needs such as food, clothing, and medicines for dependent elderly persons; *sexual mistreatment*, or being blamed for an unusual relationship with a stranger and being forced to talk about sex; and *financial mistreatment*, or the misappropriation of elders’ belongings, such as property, gold, and silver [13]. The self-reported responses were coded as ‘yes/no’ for each of the questions in each mistreatment subtype domain.

### Covariates

The following covariates were included in the study: age, sex, religion, ethnicity, educational attainment, employment status, income level of family/caregiver (*family/caregiver represents elders’ children and immediate families*), dependence on family members or caregivers for daily living activities, living arrangements, smoking habits, drinking habits, morbidity status, use of medications for any morbidity, and concentration problems. In particular, religion refers to "*a system of faith or prayers adopted by elders*". Additionally, ethnicity refers to the "*state of belonging to a social group with regard to local cultural tradition*", educational attainment refers to the "*education status of elder (yes/no)*", employment status is defined as the "*past/present employment history of elder*", living arrangement refers to "*the familial and immediate familial relationships of an elder to all the other people with whom they usually reside*", and dependence of daily living activities on caregivers/family members refers to "*routine actives of elders*, *such as eating*, *bathing*, *dressing*, *toileting*, *taking medicine*, *walking and going to the doctor*, *which are all tasks for which they have to depend on caregivers/family members*". Smoking and drinking habits are defined as the "*consumption of any forms of tobacco and alcohol in the last week*", morbidity refers to "*a type of disease self-reported by the elder at the time of the survey*", taking medication for any morbidity is defined as "*consuming a drug or other form of medicine that is used to treat or prevent disease at the time of the survey*", concentration problems indicate "*failing to recall the position of objects and forgetting to perform activities in time if instructed by family members or caregivers in the last 30 days*".

### Statistical analyses

The statistical analyses were conducted using the Statistical Package for Social Sciences (SPSS 15.00). We assessed multicollinearity of covariates using Variance Inflation Factors (VIFs). The VIFs for all covariates that were included in the logistic regression analysis were less than 2.0. Associations between the covariates and elder mistreatment were checked using binary logistic regression. The covariates that were significantly associated (p-value ≤ 0.05) with the dependent variables in univariate analysis were considered in the multivariable analysis.

## Results

### Study sample characteristics

Among the 339 study participants, 53.4% (n = 181) were males and 46.6% (n = 158) were females. The ages of the male participants ranged from 60 to 110 years, with a mean age of 71.03 (±8.85) years. Similarly, the ages of the female participants ranged from 60 to 115 years, with a mean age of 69.89 (±8.19) years. The proportion of married study participants was 67.3%, while 32.7% were separated or practiced polygamy. Of the total sample, more than half of the participants were illiterate (55.8%), and 44.2% were literate. Hinduism was the major religion acknowledged (96.2%), followed by Islam (2.9%) and Christianity (0.9%). Over 65.0% of the study participants revealed that they were employed in a private or government job. The prevalence of smoking and alcohol use was found to be 17.7% and 12.4%, respectively. Most of the elders lived in joint families (62.2%), and 37.8% lived in a nuclear family [Table 1].

**Table 1:** Socio-demographic profiles of study participants (n = 339).

Variables	Frequency	Percentage
Gender		
Male	181	53.4
Female	158	46.6
Age category(in years)		
60–74	252	74.3
75–84	67	19.8
≥85	20	5.9
Marital status		
Married	228	67.3
Separated/unmarried/polygamy	111	32.7
Ethnicity		
Upper caste (Brahmin/Chhetri)	160	47.2
Indigenous caste	122	36.0
Dalit (backward class in the traditional Hindu caste system)	57	16.8
Religion		
Hindu	326	96.2
Islam	10	2.9
Christian	3	0.9
Educational status		
Educated	150	44.2
Illiterate	189	55.8
Monthly income of family/caregiver		
≤20,000	246	72.6
21,000–30,000	48	14.1
≥31,000	45	13.3
Living arrangement		
Daughter and son-in-law	10	2.9
Son and daughter-in-law	203	59.9
Spouse/relatives	126	37.2
Current practice of smoking		
Yes	60	17.7
No	279	82.3
Current practice of drinking alcohol		
Yes	42	12.4
No	297	87.6

### Prevalence of mistreatment and subtypes of elder mistreatment

The prevalence of mistreatment was examined for the total sample and by gender. For the total sample, 61.7% reported they had experienced some form of mistreatment in the last three months. The prevalence of the most common subtypes of self-reported physical mistreatment, psychological mistreatment, caregiver neglect and financial mistreatment was 2.4% (95% CI: 0.7%-4.0%), 22.4% (95% CI: 17.9%-26.8%), 57.5% (95% CI: 52.2%-62.8%) and 12.1% (95% CI: 8.6%-15.5%), respectively. Notably, 8.3% (95% CI: 5.3%-11.2%) of elders reported mistreatment by strangers, including sexual mistreatment and mistreatment at health facilities and public transportation locations [Table 2].

**Table 2:** Gender-stratified prevalence of various types of elder mistreatment.

Types of mistreatment	Prevalence	Prevalence		Overall	95% CI
	(%) among		(%)	prevalence	
	females	among males		(%)	
Any mistreatment	65.2	58.6		61.7	56.5–66.8
Physical mistreatment	3.2	1.7		2.4	0.7–4.0
Psychological	24.7	20.4		22.4	17.9–26.8
mistreatment					
Family/caregiver neglect	62.0	53.6		57.5	52.2–62.8
Financial mistreatment	15.8	8.8		12.1	8.6–15.5
Stranger mistreatment	7.0	9.4		8.3	5.3–11.2

### Factors associated with mistreatment and subtypes of elder mistreatment

We reject the hypothesis that there is no effect of dependence on family/caregivers for daily living activities (DLA), education status and concentration problems on any type of mistreatment, after adjusting for sex and ethnicity. Similarly, we reject the hypothesis that there is no effect of living arrangement, use of medication for any morbidity, monthly income of family/caregiver, ethnicity and concentration problems on subtypes of mistreatment, after adjusting for sex and education status. However, we accept the hypothesis that there is no association between gender, age group, use of alcohol, and smoking on any type of elder mistreatment.

Table 3 presents multivariable regression results for the predictors of some form of mistreatment. The results (adjusted for demographics) showed that many elderly victims of some form of mistreatment depended on family/caregivers for daily living activities (OR = 2.26, 95% CI: 1.38–3.69), were illiterate (OR = 1.69, 95% CI: 1.01–2.82) and experienced concentration problems (OR = 1.94, 95% CI: 1.19–3.16). More specifically, elderly people who mentioned living arrangements with sons/daughters-in-law experienced financial mistreatment (OR = 2.76, 95% CI: 1.22–6.20). The variables associated with the risk of caregiver neglect were being illiterate (OR = 1.69, 95% CI: 1.01–2.83) and experiencing concentration problems (OR = 2.03, 95% CI: 1.25–3.28) in the last 30 days. Similarly, elderly people who reported taking medication for any morbidity (OR = 1.82, 95% CI: 1.01–3.29), staying with family/caregivers having a monthly family income of < NRs. 20,000 (193 USD) (OR = 3.01, 95% CI: 1.09–8.31) and coming from the Dalit community, a backward caste according to the Hindu traditional caste system (OR = 2.36, 95% CI: 1.17–4.75), were at more risk of psychological mistreatment [Table 4].

**Table 3:** Stepwise multivariable logistic regression analysis for some form of elder mistreatment and its subtypes among the elder population.

Variables	Any mistreatment OR (95%CI)
Depending on family/caregivers for Daily living activities	
No	1
Yes	2.26(1.38–3.69)
Education status	
Educated	1
Illiterate	1.69(1.01–2.82)
Having concentration problems	
No	1
Yes	1.94(1.19–3.16)

**Variables included in the model were** ethnicity, education, marital status, gender, living arrangement, employment, and dependency on daily living activities, exercise performance, concentration problems, and medication for any disease.

**Table 4:** Stepwise multivariable logistic regression analysis for mistreatment subtypes among the elder population.

Variables	Subtypes of elder mistreatment OR(95%CI)		
	Psychological	Caregiver neglect	Financial
Living arrangement			
Daughter and son-in-law	—-	—-	1.63(.18–14.59)
Son and daughter-in-law	—-	—-	2.76(1.22–6.20)
Spouse/relatives	—-	—-	1
Education status			
Educated	—-	1	—-
Illiterate	—-	1.69(1.01–2.83)	—-
Taking medication for any morbidity			
No	1	—-	—-
Yes	1.82(1.01–3.29)	—-	—-
Monthly income of family/caregiver**			
≤20,000	3.01(1.09–8.31)	**—-**	**—-**
21000–30000	1.22(0.31–4.71)	**—-**	**—-**
≥31,000	1	**—-**	**—-**
Ethnicity			
Upper caste (Brahmin/Chhetri)	1	**—-**	**—-**
Indigenous caste	0.95(0.50–1.83)	**—-**	**—-**
Dalit (backward class in the traditional Hindu caste system)	2.36(1.17–4.75)	**—-**	**—-**
Experienced concentration problems			
No	—-	1	**—-**
Yes	—-	2.03(1.25–3.28)	**—-**

The variable included for multivariable analysis model were: Ethnicity, Education, Marital status, gender, Living Arrangement, Employment, and Dependency on Daily Living activities, Exercise performance, Concentration problems, and medication for any disease.

** 1 USD = 104 Nepalese Rupee (Available from: https://www.nrb.org.np/fxmexchangerate.php, April 2017)

## Discussion

Elder mistreatment is still under recognized by policy makers and implementers and the general public. Elder mistreatment is a violation of basic human rights, and various exceptionally complex underlying factors contribute to its occurrence. Elderly adults are not only vulnerable to mistreatment, but they also often experience mistreatment because of progressive dependence, worsened function and poor health, feelings of helplessness and loneliness, and lack of support from caregivers. There is a scarce amount of information on elder mistreatment in the rural context of Nepal. To our knowledge, this is the first community-based study examining the prevalence of elder mistreatment and its risk factors in a rural setting of Nepal.

Our findings show that elder mistreatment was common, with an estimated prevalence of 61.7% in the last three months; this estimate is the aggregate of caregiver neglect and caregiver-inflicted physical, psychological, financial and other type of mistreatment. The present estimate was found to be higher than that reported in a study conducted by Yadav et al. (49.1%) in an urban setting of Nepal [13]. Similarly, the estimate is higher than that reported in India (50.0%), Europe (20.0%), or the United States of America (10.0%) [17–19]. This difference might have arisen because self-reported mistreatment is largely based on self-perception, and we did not attempt to validate elders’ reports of mistreatment with those of their caretakers because of ethical issues. Elder mistreatment continues to be thought of as taboo, mostly under-reported and ignored across the world. In this study, we tried to explore the mistreatment types by giving various examples related to the local Nepalese context in the absence of family members or caregivers. This approach might have contributed to the high prevalence of mistreatment reported in this study. On the other hand, we have excluded elders with cognitive impairment (CI) and this might have an effect on under-estimation of elder mistreatment because CI itself is a risk factor for mistreatment. Also, it is possible that some caregivers deliberately report CI to avoid interviews/ investigation.

The findings of the current study revealed that family/caregiver neglect and psychological and financial mistreatment were the most common forms of elder mistreatment, consistent with findings from other settings [12, 20–23].

Overall or subtypes of mistreatment were more commonly reported among females compared to males, but the association was not found to be statistically significant. In the present study, we found that being dependent on family/caregivers for daily living activities, being illiterate and having concentration problems alone significantly increased the likelihood of elder mistreatment. Similarly, we found that different risk factors varied by mistreatment subtypes, even after controlling for the confounders.

Elders depending on family members/caregivers for daily living activities experienced more than twice the mistreatment of those who do not depend on family members/caregivers, which may be related to the poor socio-economic or socio-cultural class of the family/caregiver. There is a strong belief in the cohesion between family members/caregivers and elders in Nepal because of socio-cultural aspects, but this cohesion does not always occur because of the major presence of Western culture and a lack of social responsibility among family members/caregivers. Most of the time, families care for elders if they have some belongings in their name or if they opt to stay in the nuclear family. Additionally, in Nepal, family members/caregivers assume the daily activities of elders as an additional job in their personal daily routine, which might lead to increased conflict at the family level, which in turn puts elders at risk of mistreatment. This circumstance also reflects the poor health of the elders, which makes them incapable of performing activities for themselves. Furthermore, the economic dependence of an elderly person on family members/caregivers for accommodation and financial support to access health services could be a source of conflict among family members/caregivers. This dependence may also increase the risk of mistreatment. These findings are consistent with findings from similar studies [24,25].

Similarly, more than half of the participants who were illiterate experienced a greater risk of mistreatment. This finding likely reflects the fact that family members/caregivers might not be aware of the impact of negligence on the health of elders or might have the perception that illiterate people cannot access legal services easily. However, there are no strong rules and regulations for elder mistreatment at any setting in Nepal, and even if such mistreatment is reported to the police, they ask the family to solve the dispute at a personal level, as this is easily considered a family matter. The evidence suggests that education can improve individuals’ economic status and increase one’s likelihood of being respected [26–29]. This is an interesting finding, given that there is a link between education status and the experience of mistreatment in the last three months. Therefore, the results show indications of a high prevalence of mistreatment among illiterate individuals in rural Nepal.

Experiencing concentration problems with the resultant inability to complete activities of daily living increase the risk of mistreatment, which is consistent with evidence from low- and middle-income countries, including Nepal [13,29,30]. Elders who were on medication for any morbidity experience psychological mistreatment more often than their counterparts, which may also be partly due to high social and economic dependence on the caregiver. In the Nepalese context, health insurance is not used, and if elders develop morbidities, caregivers/family members often manage the expenses from their own pocket or by selling the elders’ or their own belongings, which in turns puts elders at risk of verbal mistreatment directly or indirectly (e.g., using slur words, not purchasing their medicine on time). Furthermore, we found that two demographic variables, namely, monthly income level of the family members/caregivers < NRs. 20,000 (193 USD) and elders being part of the Dalit community, presented an increased risk of psychological mistreatment to elders compared to the reference group. The one potential explanation could be poor socio-economic and socio-cultural status of the family/caregiver. For instance, a caregiver with low socio-economic status might not be able to fulfill the demanding needs of elders. Furthermore, the poor socio-cultural environment of disadvantaged ethnic groups may result in caregivers’ unwillingness to listen to the needs of the elders, which may be related to elders’ increased perceived psychological mistreatment [13,19,28–33]. However, several aspects of ongoing societal change are associated with increased elder mistreatment. Nepal, while culturally and socially diverse, is one of the poorest countries in South Asia, where two out of three Nepalese individuals live in poverty. These circumstances result in societal changes, such as changes in lifestyle-related behaviors, personal characteristics, the perception of both male and female elders as burdens, the erosion of social values in the name of modernization, and a lack of trust and understanding among family members. Lifestyle behaviors, including alcohol use disorder, drug use, and personal challenges such as aggression and stress, within the caregiver/family environment might increase the risk of elder mistreatment in Nepal.

In addition, more than half of the participants being accommodated by their son(s)- or daughter(s)-in-law reported significant financial mistreatment. In Nepal, caregiving responsibilities are traditionally borne by immediately family members and most often by son(s)- or daughter(s)-in-law. In the Nepalese context, elders are either completely or to a large extent dependent on family members, particularly on son(s)- or daughter(s)-in-law, for their daily living activities. In return, they expect to receive the belongings of elders, such as cash, jewelry, and land. However, if elders refuse to promise or transfer these belongings, it could subsequently lead to greater financial mistreatment against them. This situation could explain why son(s)- or daughter(s)-in-law are often reported as the perpetrators of mistreatment in Nepal. It is well documented in the literature that the daughter-in-law is the main perpetrator of mistreatment, very closely followed by the son [17, 19, 31, 34]. Similarly, the study by Yadav et.al (13) from urban Nepal reported that staying with son(s)- or daughter(s)-in-law increases the risk of psychological mistreatment. In contrast, studies suggest that living with immediate or supportive family members is protective against elder mistreatment [35, 36]. Therefore, it is reasonable to expect that living arrangements differ across countries and socio-cultural settings, and perpetrators vary according to the local context. A detailed examination of the differential role of living arrangements with regard to elder mistreatment needs to be further explored in the Nepalese setting. Based on our findings, there are few plans of action and little awareness of aging issues, and there is a major difference in this regard between the high income and low-income countries like Nepal.

Despite the strengths of this study, it also has some limitations. The study enrolled participants from only 3 VDCs of a one rural district in Nepal; thus, the results cannot be generalized to a wider population. Our study solely reflects mistreatment cases self-reported by the elderly population using interviewer-administered semi-structured tools, for which the perspective of the caregivers might differ, which was not considered in this study. Moreover, response bias may have been present in the data. For example, on a yes/no response, some participants might have been biased towards responding yes (i.e., even if they had undergone a given experience long ago, they may have responded yes to indicate that they have indeed had that experience), and alternatively, others might have had a conservative response bias, i.e., responding positively only to the scenarios specifically mentioned in the questions. Further, with regard to sexual mistreatment, it was delved in the context of stranger mistreatment. Cultural taboos in the Nepalese context precluded us from probing deeper into the issue such as asking them regarding being forced into sexual acts against their will. Additionally, we considered a recall period of 3 months, which could be reconsidered in future studies, e.g., by having a 12-month time frame. Due to our funding constraints, we could not consider a lower margin of error, and the authors decided to designate a 5% margin of error out of convenience. Further, since this study was cross-sectional, the causal relationship between the dependent variables and covariates cannot be established. Our study emphasizes the need for longitudinal national-level studies on elder mistreatment in Nepal that include the perspectives of both elders and caregivers. Such studies would allow for the wider generalizability of conclusions through the adoption of both qualitative and quantitative methods. Longitudinal studies can capture both the incidence and prevalence at any point in time and can further provide strategic direction for policy makers to combat this public health issue. In addition, the data generated from longitudinal studies might help to advocate for the establishment of a referral system for cases of mistreatment at the primary level of care. Furthermore, this study provides data on elder mistreatment and associated factors from a rural setting, which could be useful in the design of implementation research with clear prevention strategies for the benefit of elder well-being.

### Conclusions

The evidence generated through our study shows elder mistreatment is very common in a rural community in Nepal. Our study reveals that 61.7% of 60+-year-olds experienced any form of mistreatment (physical 2.4%, psychological 22.4%, family/caregiver neglect 57.5%, financial 12.1%, and 8.3% mistreatment by strangers, including sexual mistreatment and mistreatment at health facilities and public transportation locations).

Addressing poor socio-economic or socio-cultural classes of family/caregivers and elders could have significant implications for improving the well-being of elders in Nepal. Integrated community-focused action research with comprehensive information must be designed to educate the community on elder issues in Nepal, including mistreatment. Senior Citizen Policy covers economic benefits, social security and honor participation, but it is not sufficient to meet the needs of the elder population. For instance, the elderly pension of NRs. 2000 (Approx. USD 19.23) per month is quite insufficient to meet their needs. The training of human resources on the diagnosis and reporting criteria for elder mistreatment and the future inception of attitude change promotion programs by governmental and non-governmental organizations could represent steps forward for the country.

## Supporting information

S1 Fig This is the S1 Fig: Flow diagram of the participants in the study. (TIF)

S1 Table This is the S1 Table: Socio-demographic profiles of study participants. (TIF)

S2 Table This is the S2 Table: Gender-stratified prevalence of various types of elder mistreatment. (TIF)

S3 Table This is the S3 Table: Stepwise multivariable logistic regression analysis for some form of elder mistreatment and its subtypes among the elder population. (TIF)

S4 Table This is the S4 Table: Stepwise multivariable logistic regression analysis for mistreatment subtypes among the elder population. (TIF)
