# Supplementary figures and images for: The time has come to eliminate the gaps in the under-recognized burden of elder mistreatment: A community-based, cross-sectional study from rural eastern Nepal
Source: PLoS One. 2018 Jun 20;13(6):e0198410. doi: 10.1371/journal.pone.0198410 (PMC6010235; doi:10.1371/journal.pone.0198410)

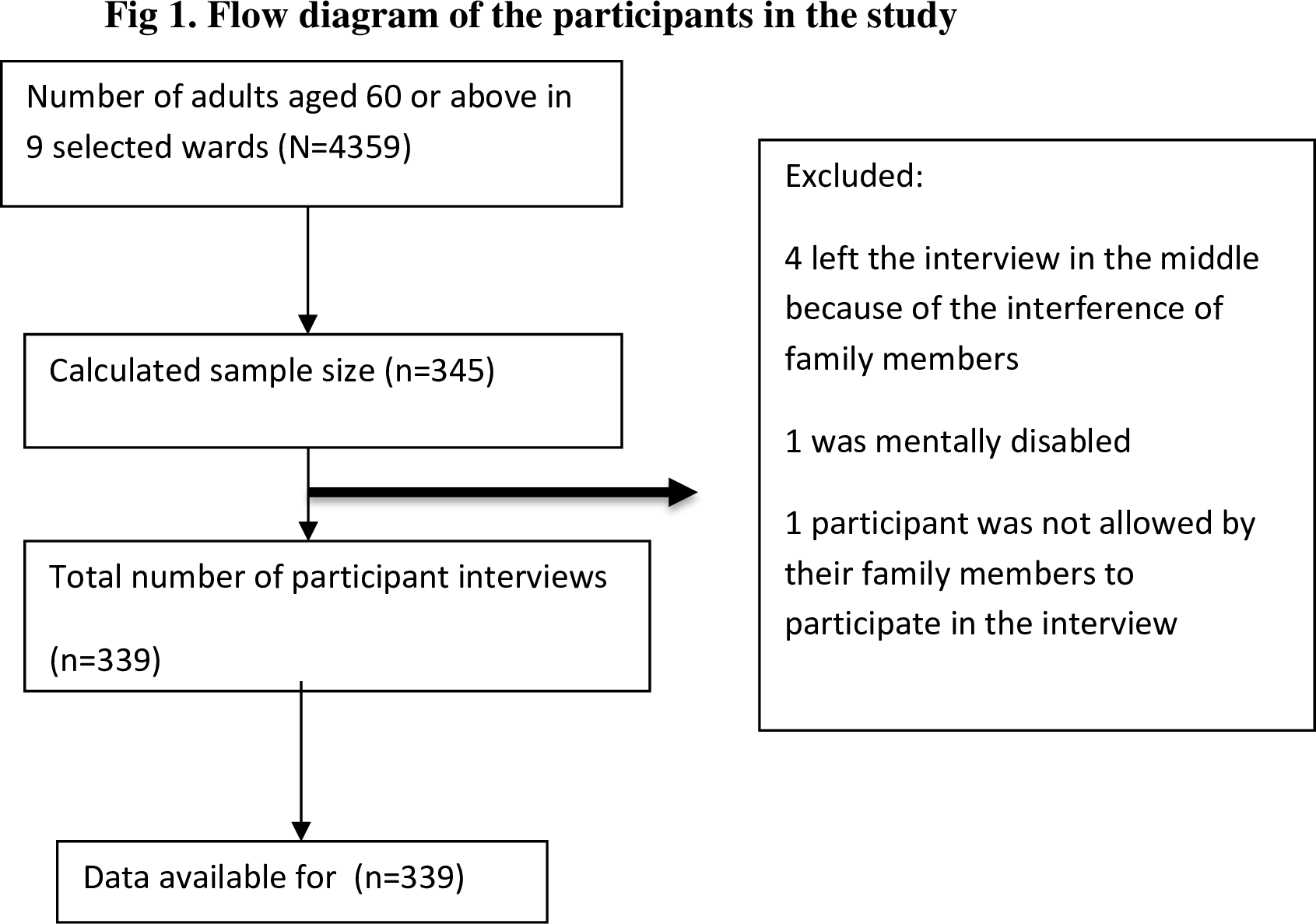

Supplement: S1 Fig — (TIF) [file pone.0198410.s001.tif]

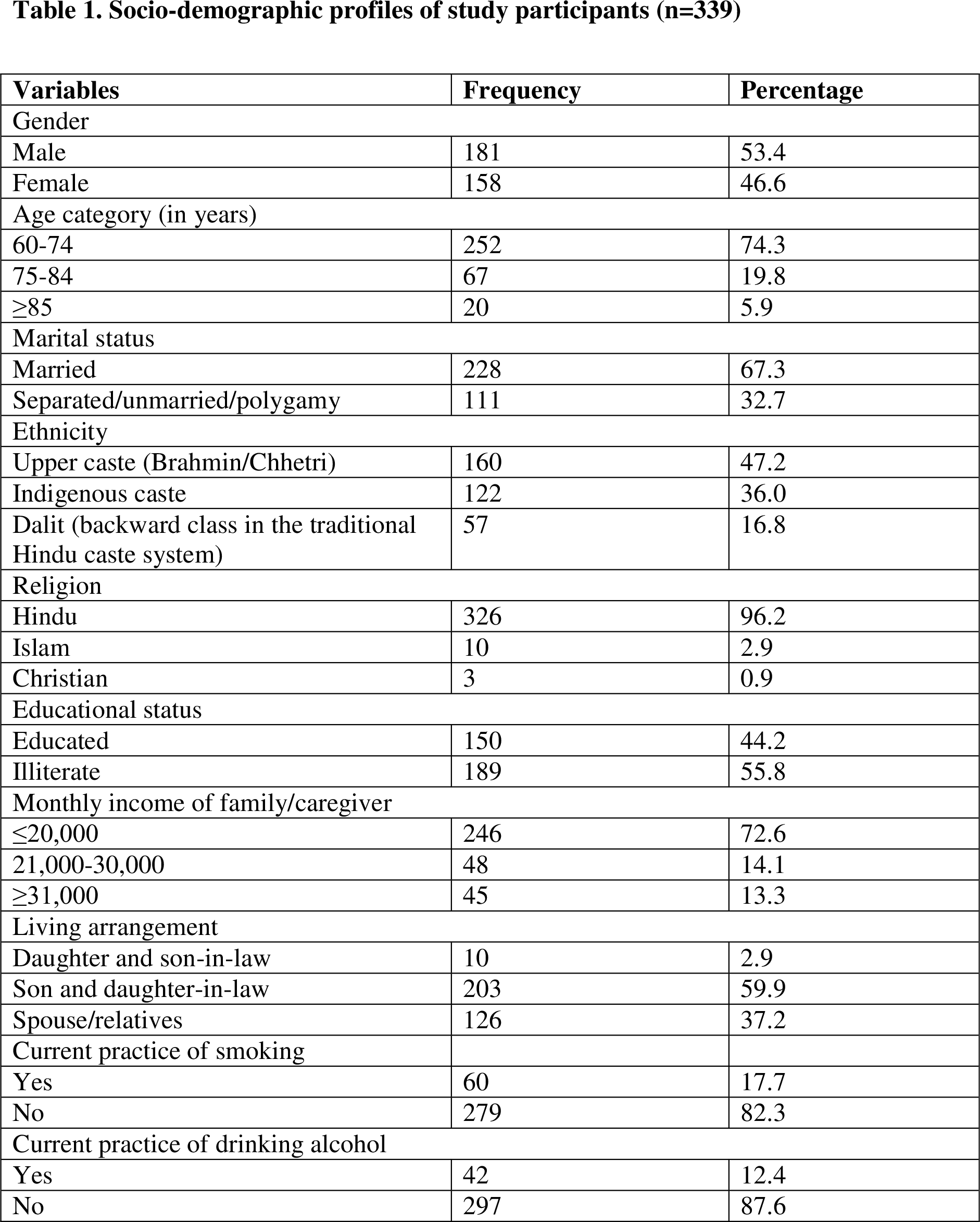

Supplement: S1 Table — (TIF) [file pone.0198410.s002.tif]

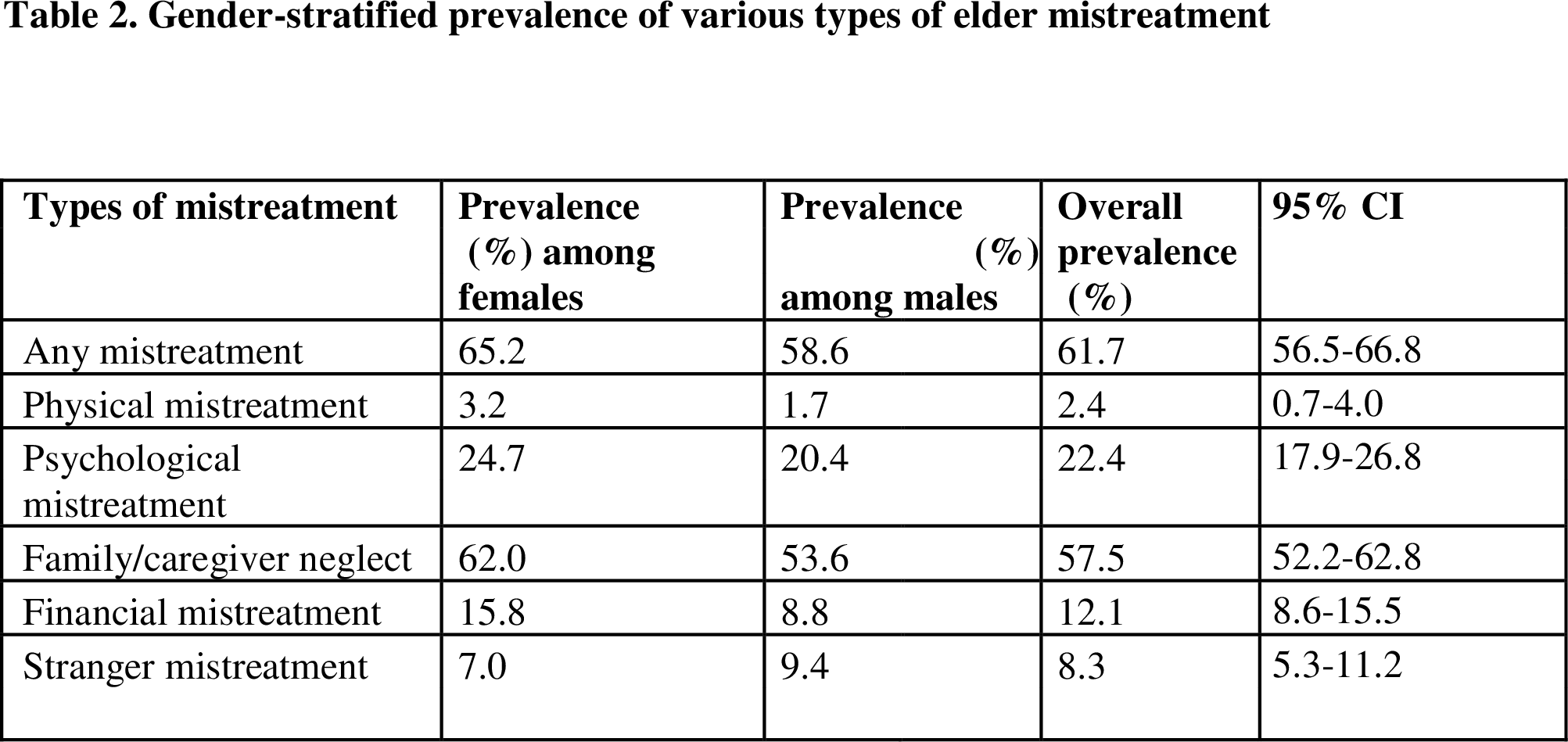

Supplement: S2 Table — (TIF) [file pone.0198410.s003.tif]

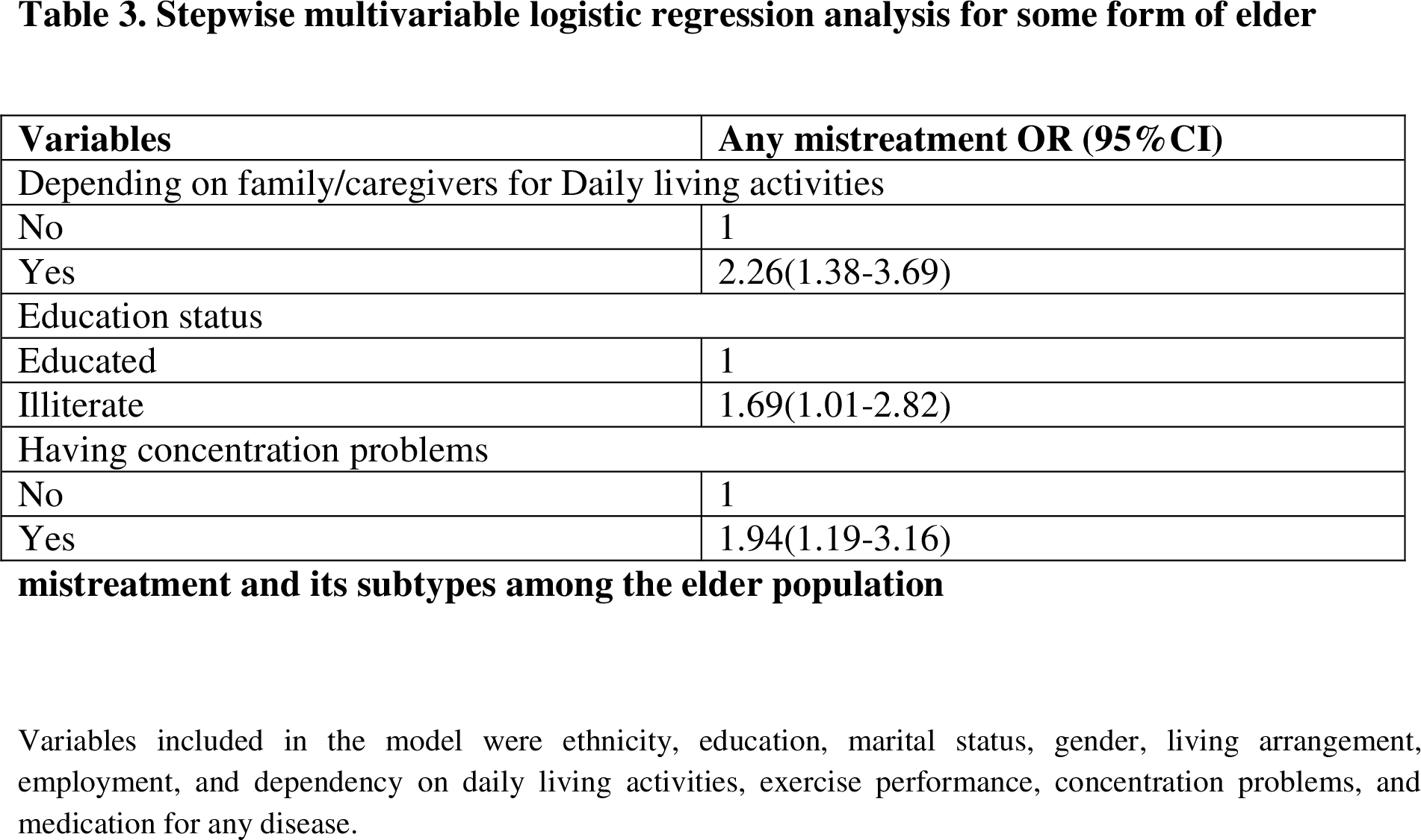

Supplement: S3 Table — (TIF) [file pone.0198410.s004.tif]

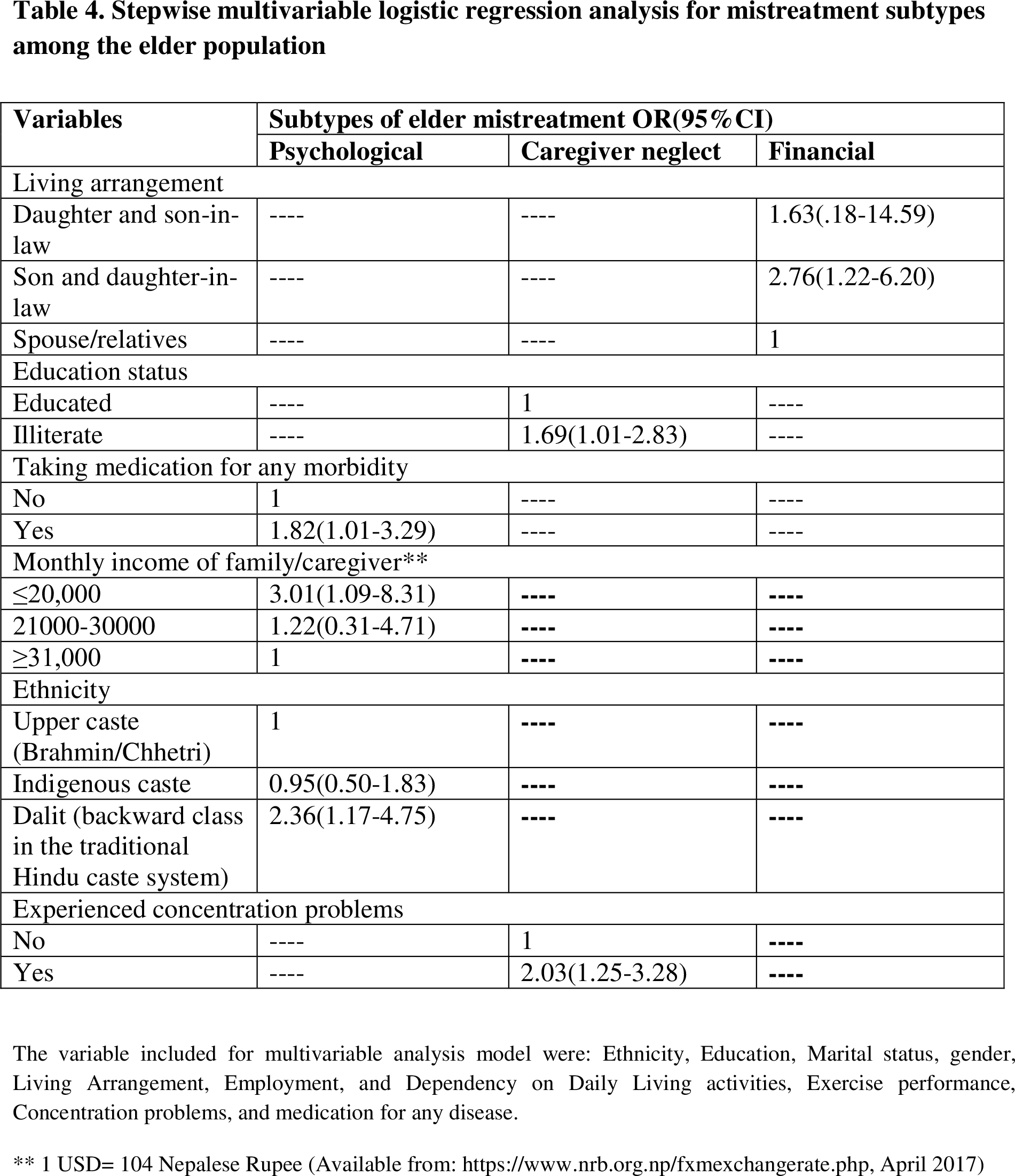

Supplement: S4 Table — (TIF) [file pone.0198410.s005.tif]
